# Repeatability and reproducibility of apparent exchange rate measurements in yeast cell phantoms using filter-exchange imaging

**DOI:** 10.1007/s10334-023-01107-w

**Published:** 2023-07-12

**Authors:** Mathias Schillmaier, Athanasia Kaika, Geoffrey J. Topping, Rickmer Braren, Franz Schilling

**Affiliations:** 1 Department of Nuclear Medicine, School of Medicine, Klinikum rechts der Isar, Technical University of Munich, 81675 Munich, Germany; 2 Diagnostic and Interventional Radiology, School of Medicine, Klinikum rechts der Isar, Technical University of Munich, 81675 Munich, Germany; 3 German Cancer Consortium (DKTK), Partner Site Munich and German Cancer Research Center (DKFZ), Heidelberg, Germany

**Keywords:** Magnetic resonance imaging, Diffusion-weighted imaging, Filter-exchange imaging, Apparent exchange rate, Transmembrane permeability, Validation phantom, Yeast

## Abstract

**Objectives:**

Development of a protocol for validation and quality assurance of filter-exchange imaging (FEXI) pulse sequences with well-defined and reproducible phantoms.

**Materials and methods:**

A FEXI pulse sequence was implemented on a 7 T preclinical MRI scanner. Six experiments in three different test categories were established for sequence validation, demonstration of the reproducibility of phantoms and the measurement of induced changes in the apparent exchange rate (AXR). First, an ice–water phantom was used to investigate the consistency of apparent diffusion coefficient (ADC) measurements with different diffusion filters. Second, yeast cell phantoms were utilized to validate the determination of the AXR in terms of repeatability (same phantom and session), reproducibility (separate but comparable phantoms in different sessions) and directionality of diffusion encodings. Third, the yeast cell phantoms were, furthermore, used to assess potential AXR bias because of altered cell density and temperature. In addition, a treatment experiment with aquaporin inhibitors was performed to evaluate the influence of these compounds on the cell membrane permeability in yeast cells.

**Results:**

FEXI-based ADC measurements of an ice–water phantom were performed for three different filter strengths, showed good agreement with the literature value of 1.099 × 10^–3^ mm^2^/s and had a maximum coefficient of variation (CV) of 0.55% within the individual filter strengths. AXR estimation in a single yeast cell phantom and imaging session with five repetitions resulted in an overall mean value of (1.49 ± 0.05) s^−1^ and a CV of 3.4% between the chosen regions of interest. For three separately prepared phantoms, AXR measurements resulted in a mean value of (1.50 ± 0.04) s^−1^ and a CV of 2.7% across the three phantoms, demonstrating high reproducibility. Across three orthogonal diffusion directions, a mean value of (1.57 ± 0.03) s^−1^ with a CV of 1.9% was detected, consistent with isotropy of AXR in yeast cells. Temperature and AXR were linearly correlated (*R*^2^ = 0.99) and an activation energy *E*_A_ of 37.7 kJ/mol was determined by Arrhenius plot. Furthermore, a negative correlation was found between cell density (as determined by the reference ADC/*f*_e_) and AXR (*R*^2^ = 0.95). The treatment experiment resulted in significantly decreased AXR values at different temperatures in the treated sample compared to the untreated control indicating an inhibiting effect.

**Conclusions:**

Using ice–water and yeast cell-based phantoms, a protocol for the validation of FEXI pulse sequences was established for the assessment of stability, repeatability, reproducibility and directionality. In addition, a strong dependence of AXR on cell density and temperature was shown. As AXR is an emerging novel imaging biomarker, the suggested protocol will be useful for quality assurance of AXR measurements within a study and potentially across multiple sites.

## Introduction

Magnetic resonance imaging (MRI) has become an integral component of routine clinical diagnostics. The technique is able to generate high contrast images, especially in soft tissue—often without the need for exogenous contrast agent application—and thus has become indispensable in the diagnosis of various diseases in all body parts and in the context of a variety of pathologies, such as spinal [1] and neurological [2] lesions, ligament injuries [3] or for the evaluation of tumour spread [4].

An even greater untapped potential of MRI is its inherent ability to visualize and quantify physiological and pathological processes on a microscopic level. An important technique herein is diffusion-weighted imaging (DWI), which is sensitive to the random translational motion of water molecules that varies between tissues depending on their composition and compartmentalization [5]. For example, DWI is highly sensitive to the detection and differentiation of cystic or metastatic lesions in the liver, where the cellular density (i.e., tissue composition) and, therefore, the ratio of fast moving extracellular water to slow moving intracellular water differs from the surrounding normal liver tissue [6, 7]. Likewise, DWI can also detect changes in compartmentalization, observed, for example, in stroke patients, where regional hypoxia leads to a breakdown of the Na^+^/K^+^-ATPase and cellular influx of water [8]. The DWI-derived apparent diffusion coefficient (ADC) quantifies the translational motion of water molecules based on the intra- and extracellular signal and is widely used in the detection and treatment evaluation in oncology [9–11].

Under physiological conditions, the intercompartment exchange of water is mediated by multiple transport proteins and channels, such as aquaporins, and has been characterized as a central component of cell physiology [12–14]. Proper function of water exchange maintains the osmolality of the cytoplasm and plays a key role in the viability of cells. Multiple studies have shown that the expression level and function of water transporting proteins is linked to the development of various pathologies, as, for example, in cancer [15], diabetes [16] or Alzheimer’s disease [17]. Non-invasive measurement of exchange processes may provide a valuable contribution to the understanding, diagnosis and treatment monitoring of diseases with disturbed membrane permeability.

Over the years, different diffusion-based techniques have been developed to quantify and visualize exchange processes [18]. Common diffusion-weighted imaging is in principle sensitive to water exchange, but the diffusion times need to be comparatively long (on the same order as the exchange processes) and the measurements need to be performed for multiple diffusion times [19–21]. In this case, the Kärger model [22–24] can be used to quantify exchange processes. However, this method is not able to fully separate exchange processes from restricted diffusion, which also needs to be taken into account at long diffusion times. Thus, exchange processes can only be estimated, which is a major limitation of this method [25, 26].

Callaghan and Furó managed to overcome this problem by implementing a technique called diffusion exchange spectroscopy (DEXSY) [27]. In this method, two pulsed gradient spin echo (PGSE) blocks are separated by a variable delay, referred to as the mixing time, while the sequence cycles through the two PGSE blocks with different diffusion weightings. The sequence is repeated for different mixing times, making it possible to detect exchange processes.

Åslund et al. refined and simplified DEXSY by replacing the variable pair of diffusion gradients in the first PGSE block with a fixed pair of gradients and introduced this technique as filter-exchange spectroscopy (FEXSY) [28]. Here, the first pair of fixed diffusion gradients acts as a filter, which is able to selectively remove the signal of the “fast-diffusing” water fraction. The subsequent return to equilibrium between intra- and extracellular fractions, at increasing mixing times, is due to exchange processes that take place during those mixing times.

A spatially resolved version of the FEXSY sequence has been developed by Lasič et al. and is called filter-exchange imaging (FEXI) [29]. Since then, the potential of FEXI has been investigated in various studies. The FEXI protocol was, amongst others, optimized for applications in the human brain, showing that FEXI may be helpful to differentiate between different types of brain tumours, [30, 31] or to detect genetically modified cells in animals via expression of aqueous pores [32]. Furthermore, multiple breast cancer cell lines were investigated using FEXSY, indicating that high exchange rates are associated with more aggressive cancer subtypes [33]. However, it was shown that in complex media involving different compartments at varying diffusivities, FEXI is also sensitive to the geometry of the involved compartments, which makes the interpretation of FEXI challenging, e.g., in grey matter in the brain [34]. Nevertheless, FEXSY/FEXI shows great potential for advanced cell and tissue characterization and possibly in the detection and evaluation of early treatment response.

Although FEXSY/FEXI has already led to very promising results, a major issue is the lack of well-defined phantoms suitable for the validation of these types of pulse sequences. Especially quality assurance protocols for assessment of reproducibility and inter-site comparability of FEXSY/FEXI measurements have not been investigated so far. Therefore, the aim of this study was to develop a protocol for the validation of FEXI implementations which is reproducible and could serve for validation across different sites. The developed validation protocol uses two types of phantoms and multiple FEXI acquisitions to assess the stability, repeatability, reproducibility and directionality dependence of the sequence implementation. In addition, experiments with varying temperatures and cell densities as well as potential aquaporin inhibitors highlight the sensitivity of FEXI measurements to these parameters.

## Materials and methods

### FEXI/FEXSY pulse sequence

**Fig. 1:**
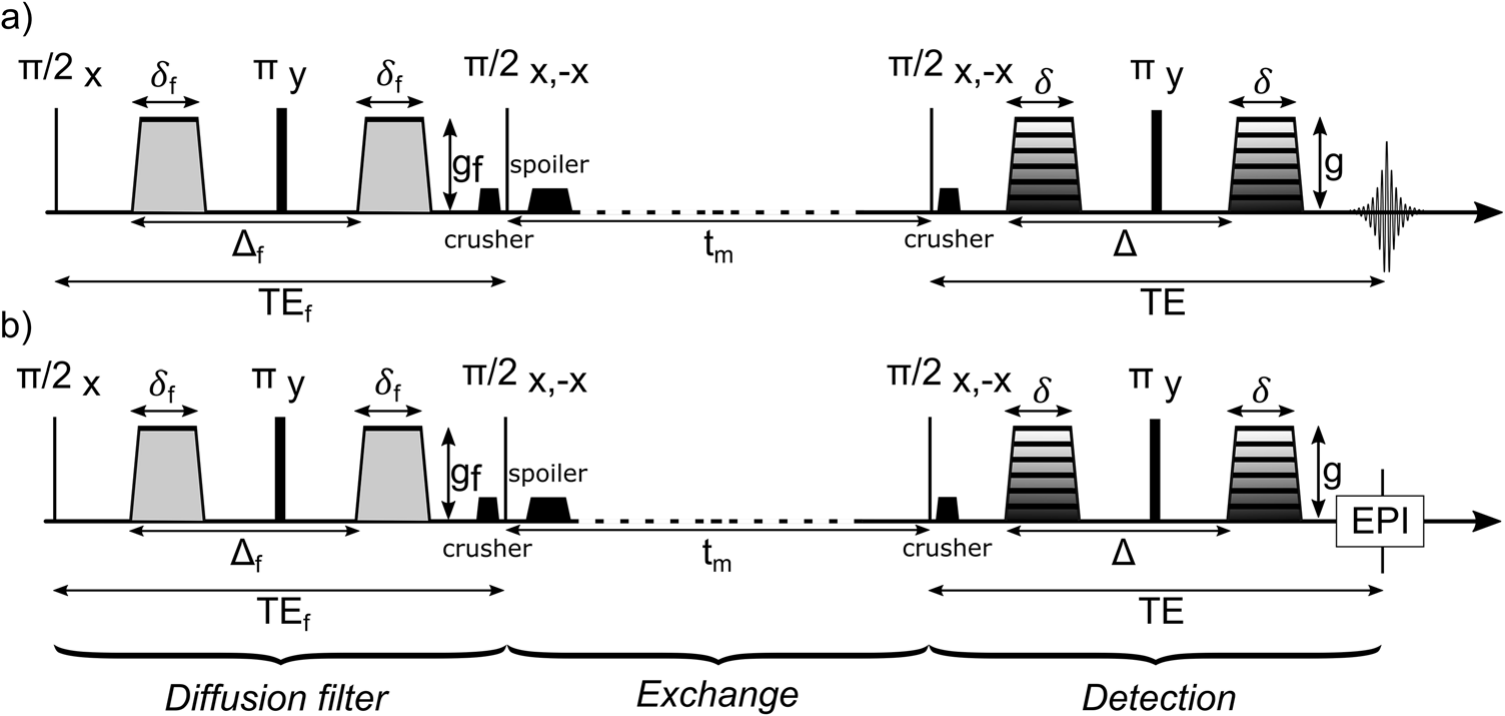
Diagram of FEXSY (**a**) and FEXI (**b**) pulse sequences consisting of three major parts: the first PGSE block, also known as filter block, is intended to suppress the fast-diffusing component in a multi-compartment system. In the second PGSE block, which is followed by an EPI read-out module in FEXI (**b**), the remaining signal is detected. The section between the two PGSE blocks represents a variable period of time, during which exchange processes can take place. This duration is also known as mixing time *t*_m_. During the mixing time, the magnetization is stored along the longitudinal axis, and a spoiler gradient directly after the second 90° pulse dephases any transversal magnetization that was excited by the second 90° pulse. Two crusher gradients (before the second 90° pulse and after the third 90° pulse) dephase and rephase the filtered magnetization, but dephase magnetization excited by the third 90° pulse. *π*/2 and *π* represent the 90° and 180° pulses. *g*_f_ stands for the filter gradient strength. *Δ*_f_ and *δ*_f_ are explained in the section “Determination of the AXR”

The FEXI sequence, first implemented by Lasič et al. [29], represents a spatially resolved double pulsed gradient spin echo (PGSE) sequence for the non-invasive determination of exchange processes across cellular membranes. The sequence (Fig. 1b) consists of three major parts: (1) A filter module, whose pair of filter gradients is intended to suppress the signal of the fast-diffusing component in a specific system (i.e., extracellular water). (2) An exchange and storage module, during which water exchange takes place, while the magnetization is stored in the longitudinal axis. This exchange period is also called the mixing time *t*_m_. (3) A detection module, consisting of a PGSE block with an EPI read out, which is similar to a standard spin-echo diffusion-weighted sequence. The FEXSY sequence (Fig. 1a) represents the spectroscopic version of the FEXI sequence lacking gradients for spatially resolved excitation and the EPI read out.

In our FEXI/FEXSY implementations, an experiment consists of two individual measurements. For the first measurement, a so-called reference scan is acquired: the FEXI/FEXSY sequence is run, while the filter gradients are turned off and *t*_m_ is set to a preferable low value. This procedure corresponds to a conventional DWI measurement (besides some additional *T*_2_ -weighting from the filter-module), resulting in an ADC which describes the average diffusivity of a system including both intra- and extracellular diffusivities. This ADC is denoted as the reference ADC (ADC_ref_).

For the second measurement, the filter gradients are turned on and the sequence cycles through increasing mixing times (*t*_m_). In this setup, the two filter gradients suppress the signal of the fast-diffusing (i.e., extracellular) water molecules, while the signal of the slowly diffusing (i.e., intracellular) water molecules is mainly preserved, leading to an overall reduction of the measured ADC. Therefore, the ADCʹ(*t*_m, min_) which describes the ADC directly after application of the diffusion filter (i.e., at minimum *t*_m_ = *t*_m, min_) represents an ADC weighted towards the intracellular ADC value. During the mixing time, intracellular water molecules are able to traverse the cell membrane. The longer the mixing time, the more water molecules will be able to exchange across the cell membrane leading to a relaxation of the measured ADC towards its equilibrium value ADC_ref_.

### Determination of the AXR

For the description of exchange processes, a simplified version of a two-site exchange model is applied as described before [28–30]. We divide our system in two compartments, in which water molecules undergo either fast or slow diffusion depending on their location, either extra- or intracellular, respectively. The two compartments and their respective fractions *f*_ex_ and *f*_in_ are normalized:

1 $$f_{{{\text{ex}}}} + f_{{{\text{in}}}} = 1.$$

Water molecules in the intracellular compartment show a reduced diffusivity due to increased viscosity, hindrance and restricted diffusion as they are in close contact to cell membranes, organelles and other macromolecules [35]. In contrast, the diffusivity of extracellular water molecules is expected to be rather high, since they are not as heavily hindered by cellular and/or extracellular obstacles. This relation is reflected in a low intracellular diffusivity *D*_in_ and a high extracellular diffusivity *D*_ex_.

The signal reduction of a group of isotropically and thermally diffusing water molecules after applying different* b* values to the system can generally be described by the Stejskal–Tanner equation:

2 $$S\left( b \right) = S_{0} \exp \left( { - b \times {\text{ADC}}} \right),$$

with* S*_0_ being the signal intensity when the diffusion gradients are turned off and *b*, also denoted as the *b* value, summarizing the diffusion weighting of a sequence (e.g., ${b=\left(\gamma \delta g\right)}^{2}(\Delta -\delta /3$*)* for paired rectangular-shaped pulsed gradients with delay *Δ*, duration *δ*, strength *g*, and gyromagnetic ratio *γ*). The ADC represents the apparent diffusion coefficient that describes the average diffusivity in a two-compartment system as

3 $${\text{ADC }} = { }f_{{{\text{in}}}}^{{{\text{eq}}}} D_{{{\text{in}}}} + f_{{{\text{ex}}}}^{{{\text{eq}}}} D_{{{\text{ex}}}} ,$$

with *D*_in_ and *D*_ex_ representing the intra- and extracellular diffusion coefficients and ${f}_{\mathrm{in}}^{\mathrm{eq}}$ and ${f}_{\mathrm{ex}}^{\mathrm{eq}}$ being the intra- and extracellular fractions at an equilibrium state.

In FEXI, after a filter has been applied, the signal attenuation varies with mixing time *t*_m_ and is given by

4 $$S\left( {b,t_{{\text{m}}} } \right) = S_{{\text{f}}} \left( {t_{{\text{m}}} } \right) {\text{exp}}\left( { - b \times {\text{ADC}}^{\prime}\left( {t_{{\text{m}}} } \right)} \right),$$

where *S*_f_(*t*_m_) describes the signal intensity after the application of the diffusion filter but before the application of the detection block. The *t*_m_-dependent population weighted average and filtered ADCʹ is then given by

5 $${\text{ADC}}^{\prime}\left( {t_{{\text{m}}} } \right){ } = { }f_{{{\text{in}}}} \left( {t_{{\text{m}}} } \right)D_{{{\text{in}}}} + f_{{{\text{ex}}}} \left( {t_{{\text{m}}} } \right)D_{{{\text{ex}}}} ,$$

*f*_ex_ is also affected by the application of the diffusion filter and exchange processes taking place during *t*_m_, while obeying standard first order reaction kinetics, resulting in

6 $$f_{{{\text{ex}}}} \left( {t_{{\text{m}}} } \right) = f_{{{\text{ex}}}}^{{{\text{eq}}}} - \left[ {f_{{{\text{ex}}}}^{{{\text{eq}}}} - f_{{{\text{ex}}}} \left( 0 \right)} \right]{\text{exp}}\left( { - kt_{{\text{m}}} } \right),$$

with *k* being the effective exchange rate given by

7 $$k = k_{{{\text{in}}}} + k_{{{\text{ex}}}} ,$$

with *k*_in_ and *k*_ex_ being the forward and reverse exchange rates.

Combining Eqs. (1), (3), (5) and (6) results in

8 $${\text{ADC}}^{\prime}\left( {t_{{\text{m}}} } \right) = {\text{ADC}} \times \left[ {1 - \sigma {\text{exp}}\left( { - t_{{\text{m}}} \times {\text{AXR}}} \right)} \right],$$

whereupon *σ* is the filter efficiency, which quantifies the reduction of the ADCʹ after the signal of the fast-diffusing component has been suppressed by the application of the filter block, and takes values between 0 and 1.

From Eq. (8), *σ* is given by

9 $$\sigma = \frac{{\left( {D_{{{\text{in}}}} - D_{{{\text{ex}}}} } \right)\left( {f_{{{\text{in}}}}^{{{\text{eq}}}} - f_{{{\text{in}}}}^{0} } \right)}}{{{\text{ADC}}}} = 1 - \frac{{{\text{ADC}}^{\prime}\left( 0 \right)}}{{{\text{ADC}}}}.$$

AXR is the apparent exchange rate for a two-compartment system and is given by

10 $${\text{AXR}} = k = k_{{{\text{in}}}} + k_{{{\text{ex}}}} .$$

### MR examination

All measurements were performed on a 7 T preclinical scanner (Bruker BioSpin MRI GmbH, Ettlingen, Germany) using a 31 mm inner diameter ^1^H/^13^C dual-tuned dual-quadrature volume coil (RAPID Biomedical, Rimpar, Germany) for FEXI measurements and a ^1^H solenoid coil with 10 mm inner diameter (Rapid Biomedical, Rimpar, Germany) for FEXSY measurements. Before starting the measurements, calculation of a *B*_0_ map and a volumetric shim covering the volume of the phantom were performed using the scanner’s default adjustments. The EPI trajectory was adjusted once and reused for the following scans of the same phantom.

FEXI measurements of the ice–water phantom were performed using the following acquisition parameters: TE_f_: 37.0 ms, TE: 37.0 ms, TR ≥ : 3100 ms, averages: 5, # slices: 1, slice orientation: axial, read orientation: + *x* → − *x*, slice thickness: 3 mm, matrix: 64 × 64, FoV: 30 × 30 mm^2^, *b* values (of the diffusion detection module): 5 (72; 172; 372; 572; 822 s/mm^2^), *δ*: 4 ms (both for filter and detection module), *Δ*: 10 ms (both for filter and detection module), diffusion gradient direction (of the filter and detection module): 1/0/0 (− *x*), filter *b* values: 34; 876; 1322 s/mm^2^, spoiler/crusher gradient strength: 147.6 mT/m (*y*), spoiler/crusher gradient duration: 0.63 ms, # mixing times: 7 (4; 34; 84; 164; 244; 324; 404 ms), # dummy scans: 5, acquisition time: ~ 10 min 35 s.

FEXI measurements in the yeast cell phantom were performed using the following acquisition parameters: TE_f_: 25.3 ms, TE: 25.3 ms TR ≥ : 3100 ms, averages: 10, # slices: 1, slice orientation: axial, read orientation + *x* → − *x*, slice thickness: 5 mm, matrix: 42 × 42, FoV: 64 × 64 mm^2^, *b* values (of the diffusion detection module): 4 (72; 272; 472; 822 s/mm^2^), *δ*: 4 ms (both for filter and detection module), *Δ*: 10 ms (both for filter and detection module), diffusion gradient direction (of the filter and detection module): 1/0/0 (-*x*), # dummy scans: 5, double sampling and gradient synchronization: off, spoiler/crusher gradient strength 118 mT/m (*y*), duration of spoiler/crusher gradients: 0.95 ms, # mixing time(s) (reference scan, filter off): 1 (3 ms), filter *b* value: 1322 s/mm^2^, # mixing times (filter on): 6 (3; 63; 123; 183; 283; 383 ms), acquisition time (filter off): ~ 2 min 38 s, acquisition time (filter on): ~ 14 min 21 s. First, the reference scan was acquired with the minimum mixing time (3 ms) and the filter gradients disabled. Then, the filter gradients were enabled and the measurement was performed for the six different mixing times.

FEXSY measurements in the yeast cell phantom were performed using the following acquisition parameters: TE_f_: 21.8 ms, TE: 21.7 ms, TR: 4000 ms, averages: 1, # slices: 1, slice thickness: 5 mm, *b* values (of the diffusion detection module): 13 (35; 80; 180; 250; 350; 480; 700; 900; 1200; 1380; 1500; 2000; 2390 s/mm^2^), *δ*: 4 ms (both for filter and detection module), *Δ*: 10 ms (both for filter and detection module), diffusion gradient direction (of the filter and detection module): 0.7/0.7/0 (*x*, *y*, *z*), # dummy scans: 5, double sampling and gradient synchronization: off, spoiler/crusher gradient strength 80 mT/m (*x*, *y*), duration of spoiler/crusher gradients: 2/1 ms, # mixing time(s) (reference scan, filter off): 1 (4.5 ms), filter *b* value: 2043s/mm^2^, # mixing times (filter on): 7 (25; 55; 115; 165; 215; 265; 305 ms), acquisition time (filter off): ~ 52 s, acquisition time (filter on): ~ 7 min 48 s. First, the reference scan was acquired with the minimum mixing time (4.5 ms) and the filter gradients were still disabled. Then, the filter gradients were enabled and the measurement was performed for the seven different mixing times.

### Phantom preparation

Two different types of phantoms were used for the validation of the FEXI sequence: an ice–water phantom and a yeast cell phantom.

**Fig. 2:**
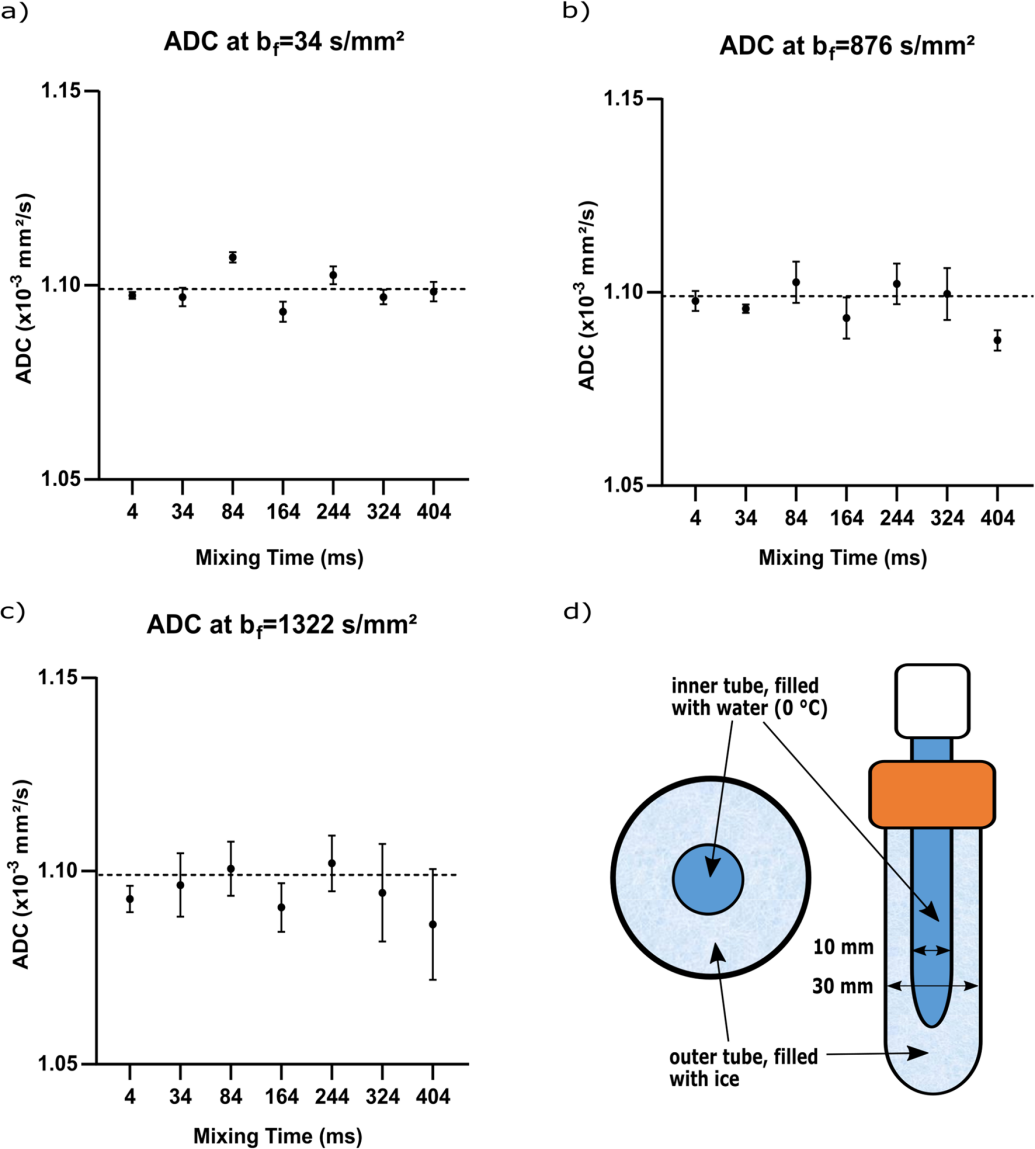
ADC stability measurements in an ice–water phantom: ADC measurements were performed at three different filter strengths (*b*_f_ = 34 s/mm^2^ (**a**), *b*_*f*_ = 876 s/mm^2^ (**b**) and *b*_f_ = 1322 s/mm^2^ (**c**) for seven mixing times with five repetitions per filter strength and mixing time. Each (**·**) indicates the mean ADC value for the five repetitions at a specific mixing time. The error bars represent the corresponding standard deviations. The dashed line represents the literature value for the ADC of water at 0 °C (1.099 × 10^–3^ mm^2^/s). **d** shows a schematic longitudinal and cross section of the ice–water phantom

The ice–water phantom (Fig. 2d) [36, 37] consists of two polyethylene tubes, one placed centrally within the other (outer diameter of the outer tube: 30 mm, outer diameter of the inner tube 10 mm). The equidistant space between the inner and the outer tube was filled with tap water, the inner tube left empty, and the whole phantom was frozen (in a standard − 20 °C laboratory freezer). Subsequently, the inner tube was filled with tap water that had been cooled to 0 °C in a freezer. The phantom was then used to measure ADCs in the water of the inner tube at a fixed temperature of 0 °C, which the outer ice ring layer maintained stably, while it melted within the MRI scanner. A stable temperature of 0 °C in the inner tube could be guaranteed as long as ice was left in the surrounding ring layer, which was checked at the end of each session.

The yeast cell phantom [28, 29, 38, 39] provides a simple isotropic two-compartment model system for the investigation of exchange processes. It consists of baker’s yeast (Saccharomyces cerevisiae), bought from a supermarket (brand: Wieninger, Germany) which has been dissolved in tap water in a 2:1 ratio (w/w yeast/water) and mixed until no large clumps of yeast were left. For the validation of the FEXI sequence and the investigation of the influence of cell density and temperature, the suspension was poured into a 50 ml polyethylene tube (a standard block of yeast of ~ 40 g and the corresponding amount of tap water are sufficient for a single phantom) and then centrifuged at 1346 g for 20 min (Thermo Scientific Heraeus Multifuge X3R Centrifuge). The centrifugation provides a large and homogeneous pellet of densely packed yeast cells, and thus prevents sedimentation processes during measurements. For the treatment experiment using the FEXSY sequence, 2 mM NiCl_2_ and 6 mM HgCl_2_ were added in the 2:1 ratio (w/w yeast/water) suspension and mixed gently at room temperature for 15 min. Then, 2.4 ml of the suspension were transferred into a NMR tube and centrifuged at 1346 g and 20 °C for 20 min. During data acquisition, an MRI-compatible temperature probe for rodents (SA Instruments Inc., Stony Brook, New York, USA) was inserted into the cell pellet for continuous temperature monitoring within the MRI scanner. An adjustable fan heater was used to keep the yeast cell pellet at controlled temperature levels by manually adjusting the air flow and temperature, based on the sample temperature displayed by the sensor.

### Experimental protocol for the validation of an FEXI sequence

Four experiments using the ice–water and yeast cell phantoms were designed to validate the implementation of the FEXI sequence in terms of stability, repeatability, homogeneity, reproducibility and directionality of AXR determination.

1. ADC stability measurements in an ice–water phantom:
  The ice–water phantom was prepared as described above. Then, FEXI scans with three different diffusion filter gradient strengths were set up and five experimental repeats were performed for every diffusion filter strength, whereupon images were acquired for five (detection) *b* values and seven mixing times. Consequently, ADC maps were calculated for every single mixing time and filter strength, separately for each repetition. First, the filter gradients were turned off to obtain a non-filtered reference scan. It needs to be mentioned that a filter *b* value of 0 s/mm^2^ is technically not achievable because of the presence of slice-selection and crusher gradients around the 180° pulse, which also contribute to the diffusion weighting. Hence, the lowest achievable *b* value in this setup is 34 s/mm^2^. Afterwards, two experiments with filter strengths of 876 s/mm^2^ and 1322 s/mm^2^ were performed to confirm a valid ADC determination at typical filter strengths used for in vivo (876 s/mm^2^) [30, 31] and in vitro (1322 s/mm^2^) [29, 33] experiments. The diffusion of water molecules within a one-compartment system is determined by the water ADC only. Thus, also after the filter application, only a reduction of the signal is expected but no changes in the estimated ADC, since exchange processes cannot take place. Measurements in this phantom are intended to ensure that the filter implementation does not bias the ADC value.
2. Repeatability and homogeneity tests in a yeast cell phantom:
  The above-mentioned yeast cell phantom was placed in the scanner and heated to a temperature of 22.5 ± 0.2 °C. The temperature was held in this range for all AXR measurements of this series. Measurements were repeated five times without removing the phantom from the scanner or re-running pre-scan adjustments (for further sequence parameters see “MR examination” section). Measurements of this phantom are intended to validate that a FEXI sequence produces repeatable results, when measuring a consistent uniform object.
3. Reproducibility test in separate but equivalent yeast cell phantoms:
  Three blocks of yeast (~ 40 g each) were solubilized in tap water in one beaker to produce a homogenous suspension of yeast cells, which was then distributed into three individual 50 ml polyethylene tubes. This procedure ensured that all three phantoms possessed the same distribution of yeast cells (as they were prepared from the same stock solution), thus forming three comparable phantoms. After centrifugation, the tubes were separately positioned in the scanner and heated to a temperature of 22.5 ± 0.3 °C. AXR measurements were repeated on each phantom five times, without moving the phantom or re-running pre-scan adjustments for that phantom (equivalent to validation experiment 2 on each). This procedure was repeated for the two remaining phantoms while keeping the temperature constant and consistent with the first phantom. These measurements are intended to validate that a FEXI sequence produces reproducible results for different but equivalent objects, and also to characterize the degree of variability in results that can be expected due to the manual phantom preparation steps.
4. Directionality test in a yeast cell phantom:
  The above-mentioned yeast cell phantom was placed in the scanner and heated to a temperature of 21.0 ± 0.1 °C. Measurements in three orthogonal gradient directions (*x*, *y* and *z*) were performed with only one repetition in each direction (since single-session repeatability had already been shown). These measurements in an isotropic cell suspension are intended to validate that a FEXI sequence produces results that do not depend on the directionality of the gradients (see Discussion).

### Influence of cell density and temperature on AXR measurements

Two additional experiments using the yeast cell phantom were performed to evaluate the sensitivity of AXR to cell density or temperature which could be a potential source of AXR bias, especially at an inter-site comparison.

1. AXR measurements at different cell densities:
  Three identical yeast cell phantoms were arranged as described in the reproducibility experiment, so that three identical cell distributions could be achieved. The different cell densities were then created by centrifuging the samples at different rotational speeds and durations: 31 min at 757 g, 20 min at 2393 g and 20 min at 4122 g. The three phantoms were then separately scanned using the FEXI technique, with five repetitions for each sample while keeping the temperature of the cell pellets at 22.5 ± 0.3 °C. Cell density/f_e_ in each sample was estimated from the ADC determined from the FEXI reference scan, for further information see “Results” section. These measurements are intended to characterize the sensitivity of a FEXI sequence to variations in the density of cells in the prepared phantom, which is an important variable in the preparation of phantoms repeatedly and at different sites. Furthermore, viability assessment of baker’s yeast cells before and after centrifugation for 20 min at 4122 g was performed with trypan blue staining and optical microscopy to ensure that centrifugation did not affect cell membrane integrity.
2. AXR measurements at different temperatures:
  A yeast cell phantom was centrifuged at 1346*g* for 20 min. Then, the phantom was consecutively measured at five different temperatures (17.2 ± 0.3 °C, 21.2 ± 0.3 °C, 25.2 ± 0.3 °C, 29.5 ± 0.3 °C and 33.3 ± 0.4 °C) with four repetitions for each temperature level, beginning at the lowest temperature. These measurements are intended to characterize the temperature sensitivity of a FEXI sequence implementation with this phantom design.

### Influence of aquaporin inhibitors on AXR measurements

Yeast cell pellets, in presence and in absence of aquaporin inhibitors, were measured at four different temperatures (19.0 ± 0.7 °C, 24.3 ± 0.2 °C, 27.0 ± 0.1 °C and 29.9 ± 0.2 °C) using the FEXSY sequence. Each sample was consecutively measured three times at an almost constant temperature. For each temperature, a new yeast cell pellet from the same batch was prepared to prevent the pellet from drying during the scan, since the sample volume was small. These measurements are meant to investigate the effect of aquaporin inhibitors on the cell membrane permeability.

### Microscopy

Viability assessment of baker’s yeast cells were performed with an optical microscope (Olympus BH2) and trypan blue staining. The intracellular volume was calculated from the cell diameter which was estimated from fluorescence microscopy (EVOS M7000 Imaging System) bright-field images. The cell cross-sectional images were analysed using the software ImageJ.

### Data analysis

For calculation of ADC values in the ice–water phantom, the signal intensities of the diffusion-weighted images from the FEXI sequence acquisitions for different *b* values were fitted to the Stejskal–Tanner equation [Eq. (2)], separately for each filter strength, mixing time and repetition. The data fitting was performed voxelwise, using nonlinear least-squares curve fitting (MATLAB, The MathWorks, Natick, MA, USA). This produced ADC maps of the phantom for every mixing time at three different filter strengths *b*_*f*_ for each of the five repetitions. Then, a region of interest (ROI) was placed on the ADC maps, covering the area of the inner tube (ROI size approx. 120 voxels), and ROI-mean ADCs (mean value of the voxelwise fitted ADC values within the ROI) for each mixing time, filter strength and repetition were calculated.

**Fig. 3:**
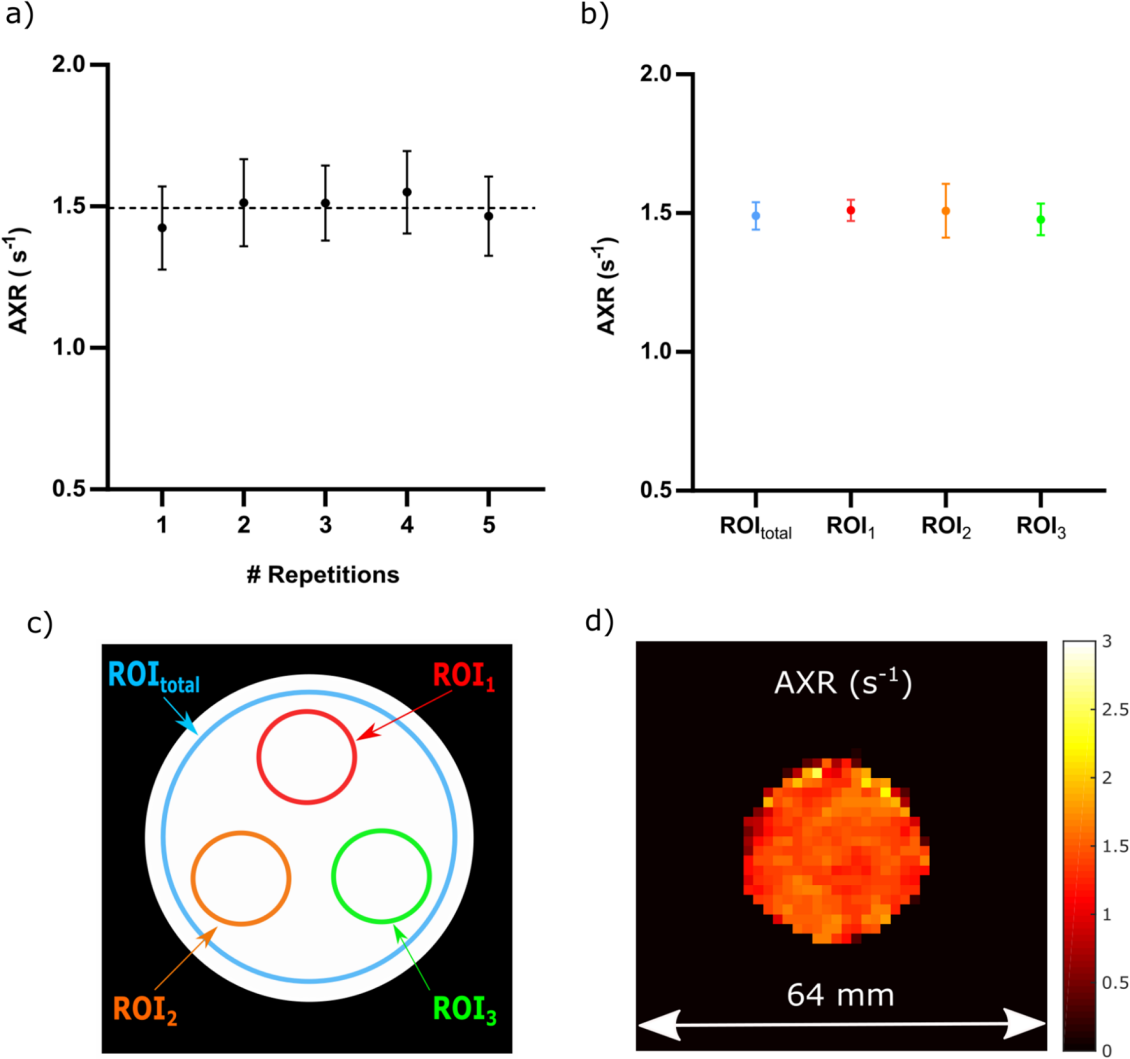
AXR map repeatability test in a yeast cell phantom: In **a**, the mean values of ROI_total_ and their corresponding standard deviations are displayed, for each of the five repetitions that were acquired, while the phantom was kept at a fixed temperature of 22.5 ± 0.2 °C. The dashed line represents the mean value across the five repetitions. **b** shows the results of the homogeneity analysis for three separate regions of interest (ROI_1_, ROI_2_ and ROI_3_) and the total ROI from (**a**) across five repetitions. Each (**·**) represents the mean value for the five repetitions of the AXR measurement for a specific ROI. The error bars indicate the corresponding standard deviations. The location of the four different ROIs is displayed in a schematic overview in **c**. **d** shows a representative AXR map acquired at a temperature of 22.5 ± 0.2 °C

For FEXI data acquired in the yeast cell experiments, ADC maps were produced as described above in the ice–water phantom for each mixing time as well as for the reference scan. Before calculating the AXR maps, ADC maps were averaged with a 2D median filter with each output voxel representing the median value of the 3 × 3 neighbourhood around the corresponding input voxel [40]. The use of the 2D median filter reduces the resolution of the images and increases the partial volume artifacts near the phantom edges. In our analyses, data of regions near the phantom edges were excluded to prevent influence on the ADC or AXR determination. Then, parametric AXR maps and the filter efficiency were calculated according to Eq. (8). AXR values within ROIs were estimated by applying the same algorithm used for voxelwise ADC fitting, but then using whole-phantom-cross-sectional ROI-averaged ADC values for the AXR determination [see blue ROI in schematic overview in Fig. 3c, ROI size approx. 190 voxels]. In addition, the homogeneity within the phantom was investigated by defining three smaller ROIs in the phantom [see red, orange and green ROIs in schematic overview in Fig. 3c, ROI size approx. 40 voxels]. In the repeatability (one data point representing one repetition) and directionality (with only one measurement per diffusion-encoding direction) experiments, the mean AXR values and corresponding standard deviations arise from an ROI [see blue ROI in schematic overview in Fig. 3c, ROI size approx. 190 voxels for repeatability experiment, ROI size approx. 175 voxels for directionality experiment] that was placed on the voxelwise fitted AXR map without averaging ADC values before performing the fit.

For FEXSY data acquired in the yeast cell experiments, the water spectral peak intensities were fitted to the Stejskal–Tanner equation [Eq. (2)], separately for each filter strength, mixing time and repetition. The data fitting was performed using nonlinear least-squares curve fitting (MATLAB, The MathWorks, Natick, MA, USA). This produced ADC values of the phantom for every single mixing time *t*_m_ and filter strength *b*_f_. Then, the AXR and filter efficiency (*σ*) values were calculated according to Eq. (8).

### Statistical analysis

Statistical analyses were performed in GraphPad Prism (GraphPad Software, San Diego, CA, USA) and MATLAB (The MathWorks, Natik, MA, USA). All results are indicated as mean value ± standard deviation (SD) or as 95% confidence interval (CI). Furthermore, the coefficient of variation (CV) (SD/mean) was calculated for the stability, repeatability, homogeneity, reproducibility and directionality experiments. In addition, a non-linear regression analysis according to the equation AXR = (*D*_ex_ − *D*_in_)/(*τ*_i_ (ADC_ref_ − *D*_in_)) (with *D*_ex_ and *D*_in_ being the two free fitting parameters and *τ*_i_ being the intracellular lifetime (which was determined from estimation of the *f*_e_ via cell counting and cell volumetry, for further information see “Discussion” section) was performed to assess the dependence between ADC_ref_/*f*_e_ and AXR, and a linear regression analysis was performed to describe the dependence between temperature and AXR in yeast cells. An analysis using an Arrhenius plot was applied to determine the activation energy *E*_A_ which represents the minimum amount of energy which is required to yield a chemical reaction (also, for example, including conformational changes in large proteins like aquaporins) [28]. Finally, an Analysis of Covariance (ANCOVA) was used to investigate whether there is a significant difference between AXR values at different temperatures and between the regression lines of treated and untreated cells, corresponding to their activation energies.

## Results

### ADC stability measurements in an ice–water phantom

ROI means of voxelwise-fit ADC maps, measured separately for seven mixing times and three filter strengths, with five repetitions per mixing time and filter strength, showed good consistency with each other and with the literature value of 1.099 × 10^–3^ mm^2^/s, which was acquired with a conventional DWI pulse sequence [41] (Fig. 2a–c). Exemplarily, the overall mean ADC values per filter *b* value across all seven mixing times (overall mean of the averaged repetitions per mixing time) resulted in [(1.099 ± 0.005) × 10^–3^ mm^2^/s, CV 0.45%] for *b*_f_ = 34 s/mm^2^, [(1.097 ± 0.006) × 10^–3^ mm^2^/s, CV 0.55%] for *b*_f_ = 876 s/mm^2^ and [(1.095 ± 0.006) × 10^–3^ mm^2^/s, CV 0.55%] for *b*_f_ = 1322 s/mm^2^ (see Table 1).

**Table 1:** Ice-water phantom mean ADCs calculated by FEXI acquisition after the application of different filter *b*-value. For each filter *b*-value, mean ADC and its standard deviation and coefficient of variation were calculated across all seven mixing times

*Ice–water phantom*			
*b*_f_ (s/mm^2^)	34	876	1322
ADC (× 10^–3^ mm^2^/s)	1.099 ± 0.005	1.097 ± 0.006	1.095 ± 0.006
CV_ADC_ (%)	0.45	0.55	0.55

### Repeatability and homogeneity tests in a yeast cell phantom

The whole-phantom-cross-sectional ROI (Fig. 3a, c) yielded an overall mean AXR value of (1.49 ± 0.05) s^−1^ with a CV of 3.4% across the five repetitions. The smaller ROIs for homogeneity assessment yielded consistent mean values of (1.51 ± 0.04) s^−1^ and a CV of 2.6% for ROI_1_, (1.51 ± 0.10) s^−1^ and a CV of 6.6% for ROI_2_ and (1.48 ± 0.06) s^−1^ with a CV of 4.1% for ROI_3_, across the five repetitions (Fig. 3b, c). The mean AXR values and their standard deviations across the five repetitions show good agreement with each other confirming repeatability of the measurements. Furthermore, the mean values of the three smaller ROIs match also well with each other and the large ROI indicating spatial AXR homogeneity. A representative AXR map is shown in Fig. 3d.

### Reproducibility test in a yeast cell phantom

**Fig. 4:**
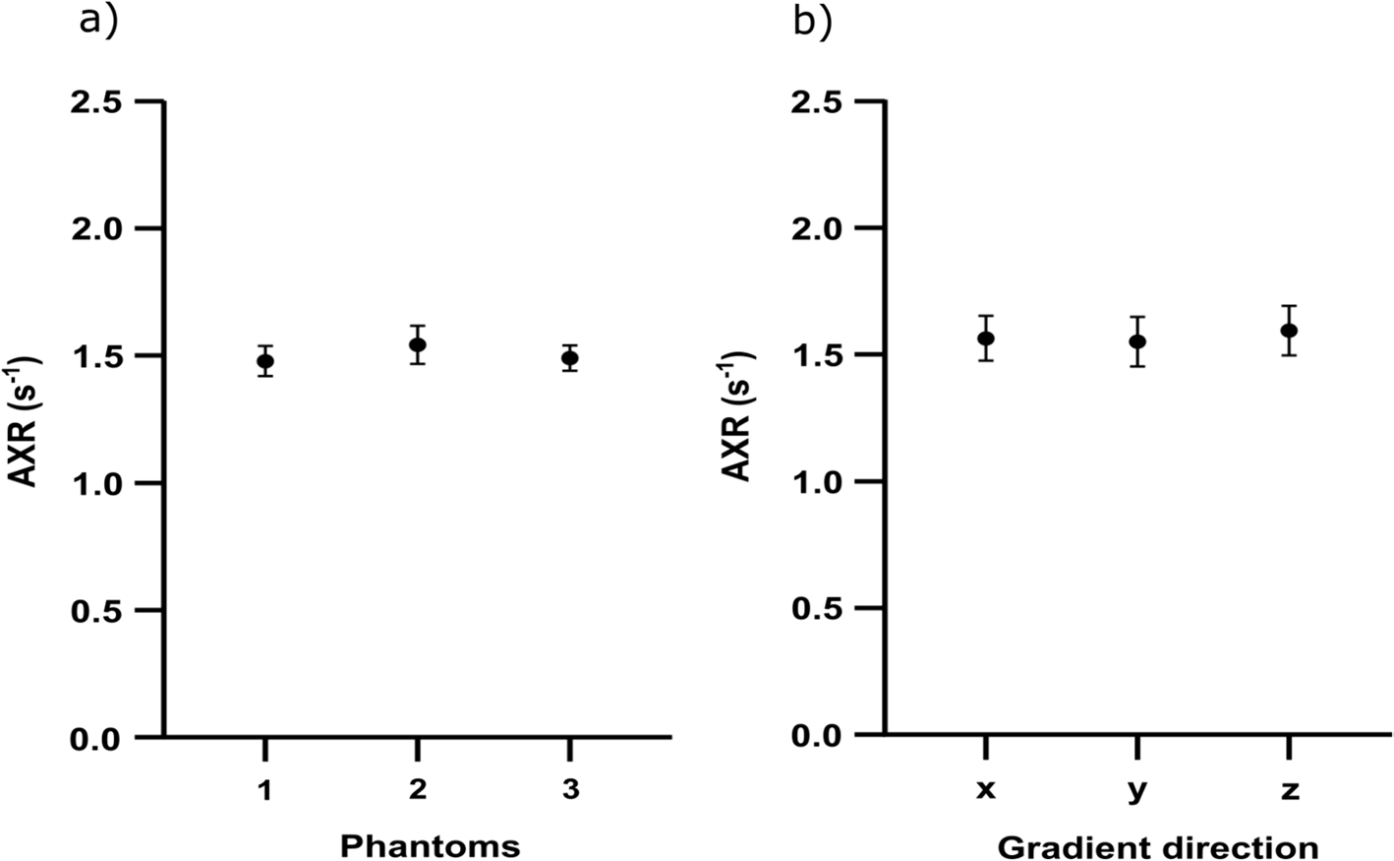
Reproducibility and directionality tests: In **a**, the results of the reproducibility experiment is displayed. Three phantoms originating from the same batch were measured with five repetitions each. Each (**·**) indicates the mean AXR value for the five repetitions. The error bars indicate the corresponding standard deviations. The directionality (**b**) was investigated for three orthogonal directions (*x*, *y* and *z*) in the yeast cell phantom. The (**·**) represents the mean AXR value within an ROI and the error bars the corresponding standard deviation for a specific gradient direction

The whole-phantom-cross-sectional ROIs, as described in the repeatability experiment, for independently prepared phantoms, yielded the following mean AXR values and standard deviations: (1.48 ± 0.06) s^−1^ for phantom_1_, (1.54 ± 0.08) s^−1^ for phantom_2_ and (1.49 ± 0.05) s^−1^ for phantom_3_, and an overall mean value of (1.50 ± 0.04) s^−1^ with a CV of 2.7% across the mean values of the three phantoms (Fig. 4a).

### Investigation of directionality in a yeast cell phantom

The AXR measured with three orthogonal gradient directions (*x*, *y* and *z*) were (1.56 ± 0.09) s^−1^ along *x*, (1.55 ± 0.1) s^−1^ along *y* and (1.59 ± 0.1) s^−1^ along *z*, with an overall mean of (1.57 ± 0.03) s^−1^ and a CV of 1.9% across the mean values of the three directions, indicating isotropy for AXR measurements in yeast cell pellets [37] (Fig. 4b).

### AXR measurements in phantoms with different cell densities

**Fig. 5:**
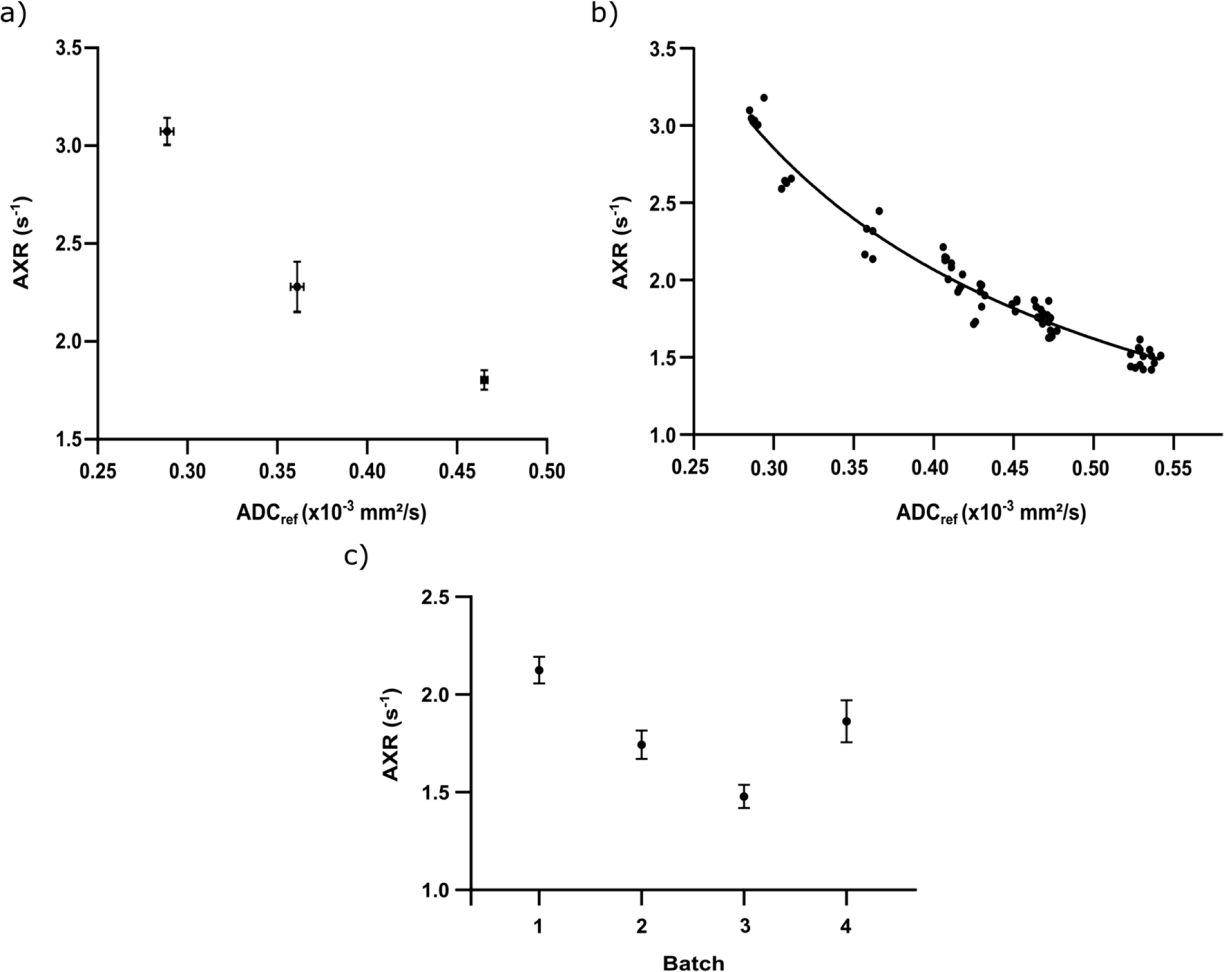
Determination of the AXR at different cell densities: In **a**, measurements in phantoms originating from one stock solution were performed at three different cell densities. Different cell densities are indicated by the (mean) ADC_ref_, resulting in high AXR values for high cell densities (low ADC_ref_) and low AXR values for low cell densities (high ADC_ref_), respectively, while keeping the cell pellet at a constant temperature. Each (**·**) indicates the mean AXR value for the five repetitions. The error bars indicate the corresponding two-dimensional standard deviations. In **b**, the plot of AXR against ADC_ref_ is displayed. Each (**·**) represents the mean AXR value within a separate ROI. The fitting model was able to accurately reproduce the correlation of AXR and ADC_ref_. For further information see “Discussion” section. In **c**, the mean values and corresponding standard deviations for four different yeast cell phantoms originating from different stock solutions are displayed

Means of the ADC_ref_ values (representing a measure of the cell density [42]) were (0.289 ± 0.004) × 10^–3^ mm^2^/s (centrifuged at 4122*g* for 20 min), (0.361 ± 0.004) × 10^–3^ mm^2^/s (at 2393*g* for 20 min) and (0.465 ± 0.002) × 10^–3^ mm^2^/s (at 757*g* for 31 min). The corresponding mean AXR values reached higher values for higher cell densities ((1.80 ± 0.06) s^−1^ vs. (2.28 ± 0.13) s^−1^ vs. (3.08 ± 0.07) s^−1^) (Fig. 5a and Table 2).

**Table 2:** Yeast cell phantom mean ADC_ref_s and AXRs. The mean ADC_ref_, mean AXR and their standard deviation were calculated across five repetitions of the same centrifugation parameters or four repetitions of the same temperature

*Yeast cell phantoms with different cell densities*						
Centrifugation	4122 g/20 min/20 °C		2393 g/20 min/20 °C		757 g/31 min/20 °C	
ADC_ref_ (× 10^–3^ mm^2^/s)	0.289 ± 0.004		0.361 ± 0.004		0.465 ± 0.002	
AXR (s^−1^)	1.80 ± 0.06		2.28 ± 0.13		3.08 ± 0.07	
*Yeast cell phantoms at different temperatures*						
Temperature (^0^C)	17.2 ± 0.3	21.2 ± 0.3	25.2 ± 0.3	29.5 ± 0.3	33.3 ± 0.4	
ADC_ref_ (× 10^–3^ mm^2^/s)	0.438 ± 0.005	0.452 ± 0.004	0.487 ± 0.007	0.522 ± 0.003	0.562 ± 0.007	
AXR (s^−1^)	1.32 ± 0.12	1.74 ± 0.11	2.23 ± 0.18	2.60 ± 0.19	3.20 ± 0.27	

Furthermore, there was also a strong negative correlation between ADC_ref_ and AXR for data sets originating from different yeast cell batches and different methods of preparation (i.e., centrifugation at different rotational speeds/pellets originating from different yeast batches), while the temperature was kept at a constant level of (22.5 ± 0.3) °C. Many of those data sets were available from multiple preliminary experiments. The data were further investigated by a non-linear regression analysis according to the equation AXR = (*D*_ex_ − *D*_in_)/(*τ*_i_ (ADC_ref_ − D_in_)) resulting in *R*^2^ = 0.95, *Sy*∙*x* = 0.0956 (standard deviation of the residuals). The fit resulted in 1.027 × 10^–3^ mm^2^/s [CI (0.9953–1.063) × 10^–3^ mm^2^/s] for *D*_ex_ and 3.716 × 10^–5^ mm^2^/s [CI (1.906–5.336) × 10^–5^ mm^2^/s] for* D*_in_ (Fig. 5b, for further information see “Discussion” section).

To show the influence of different yeast cell batches on AXR measurements, four yeast cell pellets that originated from four different batches were prepared in the same way and measured at a constant temperature of (22.5 ± 0.3) °C (six repetitions for batch 1 and 2, five repetitions for batch 3 and seven repetitions for batch 4). The results are displayed in Fig. 5c. Batch 1 yielded (2.12 ± 0.07) s^−1^ and Batch 2 (1.74 ± 0.08) s^−1^. Batch 3 resulted in (1.48 ± 0.06) s^−1^. The analysis of Batch 4 showed (1.86 ± 0.11) s^−1^.

### AXR measurements at different temperatures

**Fig. 6:**
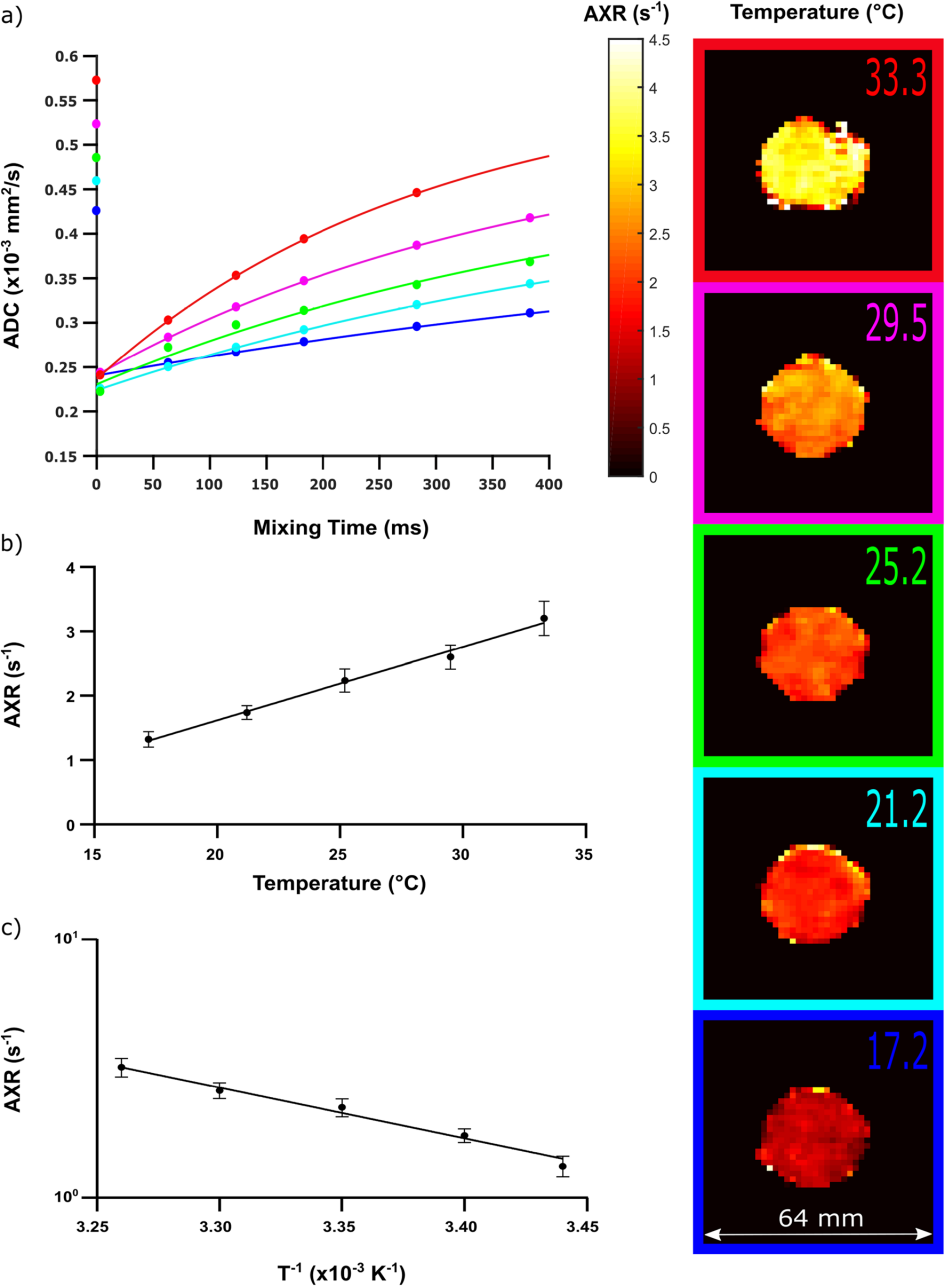
Temperature dependence of AXR measurements in yeast: In **a**, representative AXR plots for five different temperature levels are displayed. The dots represent the mean ADC_ref_ and ADCʹ(*t*_m_) values (ROI-averaged after pixelwise fitting according to Eq. (2) and the corresponding ROI had been placed in the centre of the cross section of the tube). The continuous lines indicate the fit results according to Eq. (8). For the AXR plot of the highest temperature (red line), the ADC’ (383 ms) was excluded as an outlier from further analysis (see “Materials and Methods”—> “Data Analysis”). Furthermore, five representative (voxelwise fitted) AXR maps for each individual temperature level are shown. In **b**, a linear regression analysis was performed revealing a strong linear correlation between the temperature and the AXR. Each (**·**) represents the mean value and the error bar the corresponding standard deviation of four successive measurements for each temperature step. **c** displays the Arrhenius plot revealing an *E*_A_ of 37.7 kJ/mol. As well as in **b**, each (**·**) represents the mean value and the error bar the corresponding standard deviation of four successive measurements for each temperature step

Figure 6a shows AXR relaxation curves with AXR fits calculated from the mean ADCs originating from a ROI covering the cross section of the phantom at different temperatures. In the mentioned figure, the reference ADCs (see y-axis) as well as the (filtered) ADCs’ which increase with increasing mixing times can be observed. Furthermore, there was a strong positive linear correlation between the temperature in the cell pellet and the measured AXR (*R*^2^ = 0.99, AXR = 0.114 1/(*s* × °C) × Temperature [°C] - 0.659 1/s) (Fig. 6b and Table 2). An analysis using the Arrhenius equation was conducted yielding an activation energy *E*_A_ of 37.7 kJ/mol (95% CI 29.6–46.1 kJ/mol) (Fig. 6c).

### AXR measurements in presence and in absence of aquaporin inhibitors at different temperatures

**Fig. 7:**
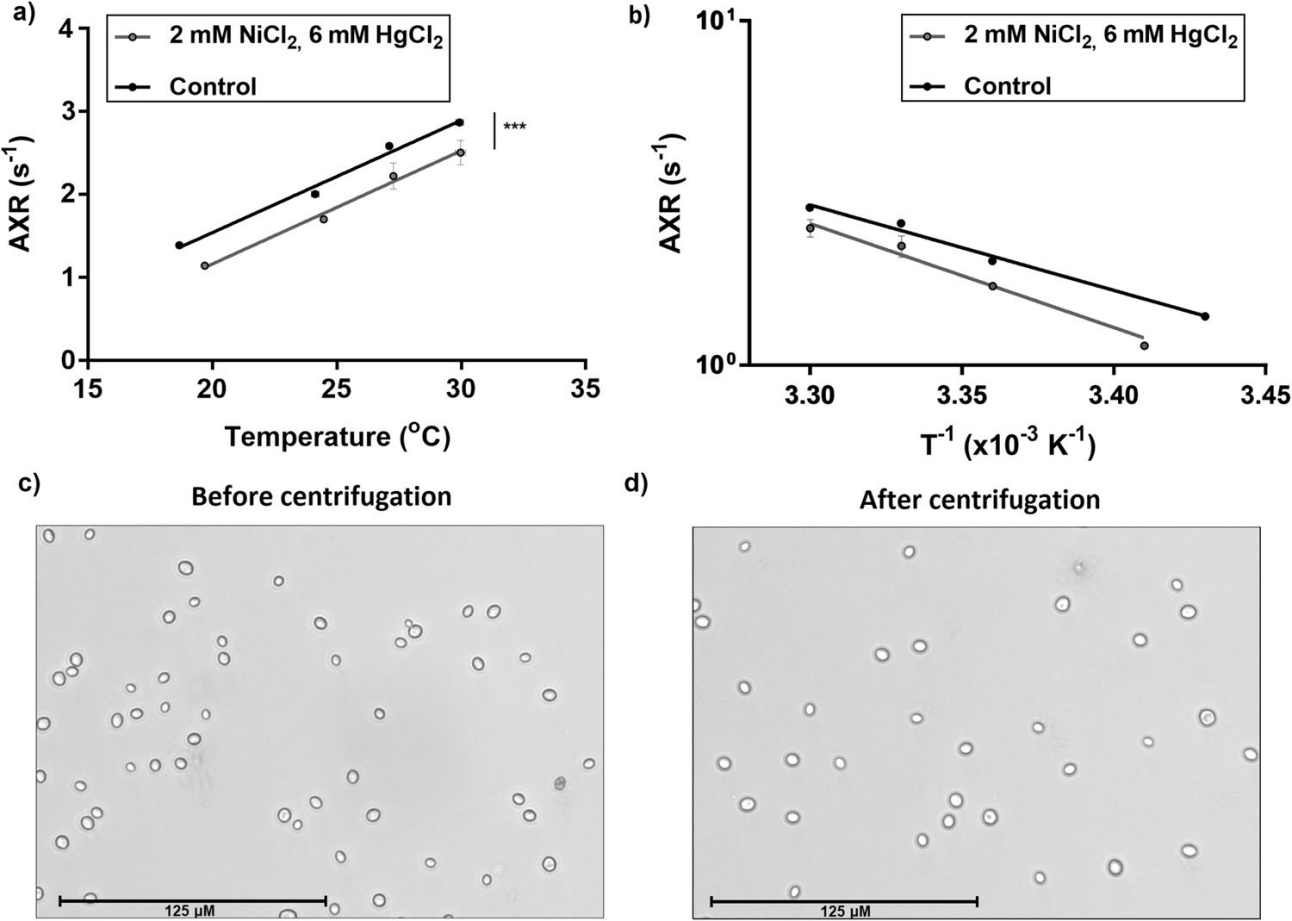
In **a** AXR values of yeast cells in presence and in absence of NiCl_2_ and HgCl_2_ as a function of temperature. The horizontal and vertical error bars of each point indicate the standard deviation over the three repetitions of the measurement of the same sample. Error bars are displayed for every single data point. For some data points, the standard deviations are so small that error bars are partly not recognisable. A linear regression analysis was performed revealing a strong linear correlation between the temperature and the AXR. An Analysis of Covariance (ANCOVA) yielded a significant difference between the elevations/intercepts of AXR values between treated and untreated cells at different temperatures (*P* = 0.0008; ***P ≤ 0.001) and no significant difference between the activation energies of treated and untreated cells. **b** displays the Arrhenius plot revealing an *E*_A_ of 47.5 kJ/mol for untreated cells and an *E*_A_ of 57.8 kJ/mol for treated cells. Error bars of each data point indicate the standard deviation over the three repetitions of the measurement of the same sample. Error bars are displayed for every single data point. For some data points, the standard deviations are so small that error bars are partly not recognisable. In **c**, **d**, representative fluorescence microscope bright-field images of yeast cells after trypan blue staining before and after centrifugation at 4122 *g* (at 20 °C for 20 min) are displayed

There was a strong positive linear correlation between the temperature in the cell pellet and the measured AXR in presence (*R*^2^ = 0.99, AXR = 0.137 1/(*s* × °C) × Temperature [°C]-1.57 1/s) and absence (*R*^2^ = 0.99, AXR = 0.136 1/(*s* × °C) × Temperature [°C]-1.17 1/s) (Fig. 7a) of aquaporin inhibitors. The AXR values of yeast in the presence of aquaporin inhibitors were lower than the AXR values of yeast in the absence of aquaporin inhibitors for all temperatures levels. Furthermore, an analysis using the Arrhenius equation was conducted yielding an activation energy *E*_A_ = 47.5 kJ/mol for untreated cells and an *E*_A_ = 57.8 kJ/mol for treated cells (Fig. 7b). An Analysis of Covariance (ANCOVA) yielded a significant difference between the elevations/intercepts of AXR values between treated and untreated cells at different temperatures (*P* = 0.0008) and no significant difference between the activation energies of treated and untreated cells.

### Cell membrane integrity after centrifugation

The percentage of vital cells after trypan blue staining was 97.5% for yeast cells which did not experience centrifugation (Fig. 7c) and 97.7%, respectively, for yeast cells having undergone centrifugation for 20 min at 4122 g (Fig. 7d).

## Discussion

The aim of this study was to establish a protocol for the validation of FEXI pulse sequences by assessment of stability, repeatability, reproducibility and directionality. Therefore, ice–water and yeast cell phantoms were chosen and adapted to test various aspects of the sequence. Special focus was put on generating reliable, robust and cost-effective phantoms that can be straightforwardly prepared.

First, a general issue in the determination of the AXR is supposed to be addressed: the above presented analysis does not consider the impact of differences in relaxation rate constants between the intra- and extracellular compartments on the AXR value. In FEXI, *T*_2_ relaxation induces signal decay during the diffusion filter and detection module and can bias the quantification of exchange if the *T*_2_ differences across the sample are ignored [43]. Eriksson et al. also suggested the combination of FEXSY with a diffusion–relaxation correlation experiment, which is able to estimate the T_2_ differences in the individual compartments and to thus eliminate its influence on the exchange estimation. In the same study, they mention that the sample preparation also affects the difference between the intra- and extracellular *T*_2_ relaxation, a fact which also needs to be taken into account when planning multi-center comparison studies.

After the implementation of the FEXI sequence, it was important to validate the reliable measurement of ADC values, since their accurate determination forms the basis of any further exchange measurements. The ice–water phantom was chosen as a simple and reproducible one-compartment model system, where no exchange processes take place. Hence, the acquired ADC values after the application of the filter gradients are not expected to differ as the filter *b* value changes (Table 1). This fact predestines the phantom for ADC stability measurements with experiments being designed to cover a wide range of FEXI-relevant parameters, such as short and long mixing times, different filter *b* values and a spatial resolution which was higher than in the subsequent FEXI experiments, to ensure that reliable and consistent ADCs can be acquired, even in extreme parameter constellations, without unexpected influence on the ADCs. Overall, good agreement of the measured mean ADC values with the literature value within their confidence intervals was chosen as criterion for a valid sequence implementation.

The yeast cell phantom represents a simple isotropic two-compartment system, where exchange processes take place and thus the measured ADC’ values depend on the filter strength and the mixing times. Initial planning experiments demonstrated the feasibility of exchange measurements in yeast cells, but also revealed major inter-experimental variations in ADC and AXR measurements, indicating the need for a highly controlled setup for reproducible phantom measurements. Variation between measurements was overcome by a meticulous control of phantom temperature (e.g., a temperature variation of ± 3 °C causes an AXR variation of ca. ± 0.34 s^−1^ according to the results of AXR measurements at different temperatures). The determination of AXR was performed in a phantom consisting of a densely packed pellet of yeast cells. The advantage of a cell pellet over a cell suspension is a noticeable lower susceptibility/sensitivity to motion artifacts caused by vibrations inside the MRI scanner during an acquisition. Furthermore, the effect of sedimentation of densely packed yeast cells over time is negligible compared to in a suspension and does not affect the measurements.

The repeatability and homogeneity tests in a single yeast cell phantom were promising, but the AXR values differed when repeating the measurements in other phantoms that had been prepared in the same way but originated from other yeast cell batches (i.e., different blocks of yeast). Figure 5c indicates AXR differences of up to 0.64 s^−1^ between the mean values of four different batches that had been prepared in the same way and measured at the same temperature. Besides the temperature, the cell density was also recognized as an important factor in AXR determination, since the density distribution between the phantoms differed (indicated by varying values for ADC_ref_), in part because commercially available baker’s yeast was used to prepare the phantoms (one cube per phantom), with which a constant cell density and cell distribution could not be guaranteed. This problem was overcome by creating multiple phantoms from the same (large) stock solution (consisting of multiple cubes of yeast dissolved in tap water). This approach finally achieved a homogenous cell density across multiple phantoms, proving that reliable and reproducible AXR measurements are possible, even for different but identically prepared phantoms (Fig. 4a). Creating a large stock solution by purchasing a large enough block of yeast or cultivating yeast cells over a longer period of time also provides the possibility to perform validation experiments at the same site at different times but also to send yeast samples from the same stock solution to different sites.

A cell pellet consisting of centrifuged yeast cells is generally considered to be isotropic, since the cells themselves are spheres/ovoids (Fig. 7c, d) without offering a preferred diffusion direction to their intracellular water molecules. Furthermore, the cells do not form any microstructures which might influence the behaviour of diffusing water molecules in the extracellular space, so that even the exchange rates should not differ between different diffusion directions. To confirm the isotropy of AXR measurements regarding different diffusion gradient directions, the apparent exchange rates were measured in three orthogonal gradient directions, which confirmed the expected directional isotropy of the AXR in an isotropic medium. These results agree well with the findings of Sønderby et al. [38], although the absolute AXR values differ, likely due to differences in temperature, cell density and sample preparation.

As mentioned above, preliminary experiments have shown that AXR values can vary substantially between two separately prepared phantoms, despite identical protocols for the sample preparation and the measurements being performed at the same temperature [44]. This effect was further investigated by preparing three phantoms with different cell densities created by varying the rotational speeds and the duration of the centrifugation process during the sample preparation prior to measurement. The ADC_ref_ was chosen as a measure of the cell density in the phantom [42], with a low ADC_ref_ indicating a high cell density and vice versa. Different cell densities finally yielded differences in the AXR, with high cell densities resulting in high AXR values and vice versa [45] (Table 2). In addition, centrifugation did not affect cell membrane integrity, indicating that that did not contribute to the high AXRs of the high cell densities (Fig. 7c, d).

With varying cell densities, the extracellular volume fraction is also altered. An increased cell density in the phantom led to a decreased extracellular volume fraction and a decreased ADC_ref_. This is consistent with Mikayama et al. [46], who showed a negative (linear) correlation between decreasing extracellular volume fraction and the ADC, indicating that the ADC and, in the case of FEXI, the ADC_ref_ are sensitive to the extracellular volume fraction. Furthermore, Lasič et al. revealed a correlation between the extracellular volume fraction and the AXR [29] as well as the intracellular liftetime *τ*_i_, summarized by the equation AXR = 1/(*τ*_i_ × *f*_e_). For further analysis of our data, the extracellular volume fraction *f*_*e*_ was approximated linearly by a two compartment approach using Eq. (1): ADC_ref_ = *f*_e_ *D*_ex_ + *f*_in_*D*_in_ =  > *f*_ex_ = (ADC_ref_ − *D*_in_)/(*D*_ex_ − *D*_in_) which was again inserted in AXR = 1/(*f*_e_ *τ*_i_) and then fitted to AXR = (*D*_ex_ − *D*_in_)/(τ_i_ (ADC_ref_ − *D*_in_)) with *D*_in_ and *D*_ex_ being the two free fitting parameters for the intra- and extracellular self-diffusion coefficients. *τ*_i_ = 1.32 s was calculated from experimental data by determination of the extracellular volume fraction *f*_e_ via cell counting and cell volumetry. Then, *τ*_i_ was calculated according to *τ*_i_ = 1/(AXR *f*_e_). The calculated value for *τ*_i_ is about twice as high as the intracellular lifetime obtained by Labadie et al. (1.32 s vs. 0.67 s) [28, 47]. However, different yeast strains, methods of sample preparation, temperatures and techniques for determination of *τ*_i_ (*T*_1_ relaxation/paramagnetic doping vs. FEXSY/FEXI) were used, so that the determined *τ*_i_ = 1.32 s seems to be acceptable for further analyses. The fitting model resulted in a good description of the existing correlation between AXR and ADC_ref_ (*R*^2^ = 0.95, *Sy∙x* = 0.0956 (standard deviation of the residuals)) (Fig. 5b). Furthermore, the data fitting of *D*_in_ and D_ex_ corresponds well with the findings of Soltesova et al. [48] who also investigated yeast cells using the FEXSY technique resulting in *D*_in_ = 1.86 × 10^–5^ mm^2^/s and *D*_ex_ = 1.04 × 10^–3^ for Saccharomyces cerevisiae at 20 °C (results of our experiments: 1.027 × 10^–3^ mm^2^/s for *D*_ex_ and 3.716 × 10^–5^ mm^2^/s for* D*_in_).

Some limitations of this analysis need to be mentioned. First, commercial baker’s yeast was used for the measurements and was not further analysed in terms of strain etc., which might play a role in determining the diffusion and exchange properties. This is especially relevant when measuring cells from different batches over time, or between sites in different cultural regions, which may have substantially different methods of yeast packaging. Studies in human cell lines by Katashima et al. [49] have shown that the centre-to-centre distance also influences the ADC, since, especially at high cell densities, this parameter can decrease below a critical value, where restricted diffusion effects could also influence the ADC measurements. In addition, for our study, effects of restricted diffusion might have affected the measurements, especially at higher cell densities, even though short echo times were used, so that the effects are expected to be relatively small.

Next, the temperature dependence of the AXR in yeast cell phantoms was investigated, revealing a strong positive correlation between temperature and AXR, which can be well-described by linear regression analysis. The results from the Arrhenius plot (yielding an *E*_A_ of 37.7 kJ/mol) also correspond well with the experimental findings of Åslund et al. [28] (40 ± 5 kJ/mol), who investigated the cell membrane permeability and activation energy in yeast using the FEXSY technique and suggest the aquaporins to be closed and the cell membrane to be the limiting factor of water exchange [28]. Soltesova et al. performed similar experiments also using the FEXSY method. Their analyses resulted in activation energies of 29.0 kJ/mol claiming facilitated water transport by aquaporins [48]. However, the results are not directly comparable, since different yeast strains (baker’s yeast vs. lab strain) were used in these experiments, and different protocols for sample preparation were applied, which might also influence the behaviour of the yeast cells. Furthermore, Pettersson et al. [50] summarized the different types of aquaporins and aquaglyceroporins being expressed in different yeast strains and under different physiological conditions. In addition, the composition of the cell membranes can also be very heterogeneous [51]. These parameters make it nearly impossible to generally determine an activation energy for yeast cell membranes. To receive comparable results highly controlled strains under highly controlled conditions need to be investigated.

Finally, a treatment experiment was performed. Two compounds which had successfully shown an aquaporin inhibiting effect in human mammalian cells [52–54] were added to a suspension of yeast cells resulting in significantly lower AXR values and a ≈ 22% higher activation energy for treated cells, whereas the difference between the activation energies was not significant. Hyperosmolarity as an explanation for the increase of the activation energy is very unlikely, since increased extracellular ion concentrations would induce cell shrinkage resulting in elevated AXRs and lowered activation energies [18]. Further investigations need to be performed but it seems plausible that the two tested substances are able to induce an inhibiting effect on water molecules traversing yeast cell membranes.

When looking at the linear correlation of temperature and AXR (see Figs. 6b, c and 7a, b), a limitation needs to be mentioned: The temperature dependencies show distinct linear correlations both in treated and untreated cells. However, only relatively small temperature ranges of ca. 10 °C and ca. 17 °C, respectively, were investigated, since we were interested in the behaviour of yeast cells in physiological temperature ranges that are easily achievable in validation experiments. In these small temperature ranges, smooth functions are often suggestive of being linear, although the global temperature dependence does not necessarily behave in a linear way. In summary, the results reflect the local behaviour of the cells for a certain temperature range, but it can neither be necessarily transferred to different temperature ranges nor to the global behaviour of the cells.

This study was intended to provide a protocol of experiments with two different types of phantoms for validation of FEXI sequence implementations. The advantage of the ice–water phantom is the existence of a well-defined ADC value at the temperature of 0 °C. This provides the possibility for single-site sequence validation but also for comparability studies across multiple sites. In contrast, the yeast cell phantom lacks a well-defined AXR value at a certain temperature, since other parameters such as strain, cell density etc. also contribute to the AXR. Nevertheless, the phantom and the proposed experiments can still be used for sequence validation even across different sites by comparing the standard deviations and/or CVs instead of the absolute AXR values. Furthermore, it is also possible (but in some cases definitely very challenging) to generate phantoms from a central stock solution which can be sent to different sites (under standard refrigerated conditions, where yeast cells stay viable for a long period of time) guaranteeing an identical cell distribution. Then, this setup may provide the chance of performing comparability studies across multiple sites, where the absolute AXR values can be compared with each other.

## Conclusions

Two different types of phantoms (ice–water, yeast cell pellet) were established for validation of the implementation of a FEXI sequence. Experiments showed high stability, repeatability, homogeneity, reproducibility and non-directionality, which might also be used for sequence validation and quality assurance across different sites. In addition, two proof-of-principle experiments were performed, inducing controlled changes in the AXR by either varying the cell density or the temperature within the phantom, resulting in increasing AXR values at low cell densities and augmenting AXR values at increasing temperature levels. Finally, a treatment experiment was performed indicating an inhibiting effect for water transport across cell membranes.

## Data Availability

The data supporting the findings of this study are available within the article or from the corresponding authors on reasonable request.
